# Recent Advances in the Diagnosis, Pathogenesis, and Management of Myxoinflammatory Fibroblastic Sarcoma

**DOI:** 10.3390/ijms25021127

**Published:** 2024-01-17

**Authors:** Jun Nishio, Shizuhide Nakayama, Mikiko Aoki

**Affiliations:** 1 Section of Orthopaedic Surgery, Department of Medicine, Fukuoka Dental College, 2-15-1 Tamura, Sawara-ku, Fukuoka 814-0193, Japan; 2 Department of Orthopaedic Surgery, Faculty of Medicine, Fukuoka University, 7-45-1 Nanakuma, Jonan-ku, Fukuoka 814-0180, Japan; n.shizuhide@gmail.com; 3 Department of Pathology, Faculty of Medicine, Fukuoka University, 7-45-1 Nanakuma, Jonan-ku, Fukuoka 814-0180, Japan; mikikoss@fukuoka-u.ac.jp

**Keywords:** myxoinflammatory fibroblastic sarcoma, diagnosis, pathogenesis, treatment

## Abstract

Myxoinflammatory fibroblastic sarcoma (MIFS) is an infiltrative, locally aggressive fibroblastic neoplasm of intermediate malignancy that typically arises in the distal extremities of middle-aged adults. It can histologically be confused with a number of benign and malignant conditions. Recently, high-grade examples of MIFS have been described. Immunohistochemistry plays a very limited role in the diagnosis of MIFS. Several genetic alterations have been identified in MIFS, including a t(1;10)(p22;q24) translocation with *TGFBR3* and/or *OGA* rearrangements, *BRAF* rearrangement, and *VGLL3* amplification. Although it appears that *VGLL3* amplification is the most consistent alteration, the molecular pathogenesis of MIFS remains poorly understood. A wide resection is considered the standard treatment for MIFS. Radiotherapy may be a viable option in cases with inadequate surgical margins or cases where surgery is likely to cause significant functional impairment. The systemic treatment options for advanced or metastatic disease are very limited. This review provides an updated overview of the clinicoradiological features, pathogenesis, histopathology, and treatment of MIFS.

## 1. Introduction

Myxoinflammatory fibroblastic sarcoma (MIFS) is an ultra-rare soft tissue tumor first described in 1998 by three independent groups under different designations, including inflammatory myxohyaline tumor of distal extremities with virocyte or Reed–Sternberg-like cells [1]; acral MIFS [2]; and inflammatory myxoid tumor of the soft parts with bizarre giant cells [3]. According to the latest World Health Organization classification of soft tissue and bone tumors, MIFS belongs to the fibroblastic/myofibroblastic tumor group [4]. The estimated incidence of MIFS is less than 1 per 1,000,000 each year [5]. Most MIFSs occur in the distal extremities of middle-aged adults. MIFS has a distinctive morphology and can be a diagnostic challenge [6]. The molecular landscape of MIFS is wide. Clinically, MIFS is characterized by a high risk of local recurrence related to its infiltrative growth pattern. Although distant metastasis is rare, high-grade variants of MIFS have recently been reported [7,8]. Complete surgical resection remains the standard treatment strategy for localized MIFS. There is currently no consensus regarding the optimal treatment strategy for advanced or metastatic disease. Large randomized clinical studies have not been undertaken for MIFS. This review highlights the clinical, radiological, histological, and genomic features of MIFS. In addition, we will summarize the current management of this ultra-rare disease.

## 2. Clinical Characteristics

MIFS has a peak incidence in the fourth to sixth decades of life (a median age of 39 years), with no gender predilection [9]. Only a few cases have been encountered in children and adolescents [10]. The etiology of this neoplasm is unknown, although a history of preceding trauma is described [9]. MIFS presents as a slowly growing, usually painless, subcutaneous mass with ill-defined margins. However, MIFS can be aggressive and rapidly grow. The mean tumor size is 3.2 cm [9]. The majority of MIFSs arise in the distal extremities, with hands and fingers being the most common sites [9,11]. The lesion shows a predilection for the dorsal aspect of the hand and foot [9]. MIFS may also occur in less common sites such as the neck, trunk, and buttock [11,12]. The symptoms at disease onset are primarily related to the site of origin. Clinically, a small nodular MIFS in the hand or foot often suggests an inflammatory process or a benign soft tissue tumor such as tenosynovitis, a ganglion cyst, or a giant cell tumor of the tendon sheath. Incomplete resection is significantly more common after primary surgery compared with revision [11].

Local recurrences occur in 22–67% of cases [1,2,9,11], often repeatedly and necessitating amputation. The median time to first recurrences is 13–15 months from the date of surgery [9,11]. Lombardi et al. suggested that, among the clinicopathological variables, symptom duration was significantly associated with the risk of local recurrence at 5 years [11]. Distant metastases are rare in conventional MIFS, with reported incidences of approximately 3% [11,13]. The lung appears to be the most common distant metastatic site for MIFS, regardless of the presenting site. In contrast, high-grade MIFS possesses high metastatic potential. Recently, Michal et al. reported that 9 (50%) of 18 high-grade MIFS patients with available follow-up developed metastases, and 7 of these patients died of metastatic disease [7]. In this case series, many tumors occurred in the proximal extremities. The median age was higher than in conventional MIFS (66 years compared with 39 years), and the mean size was larger (8.3 cm compared to 3.2 cm).

## 3. Imaging Features

A variety of imaging modalities have been applied for the detection and follow-up of MIFS. It is important to be familiar with the key imaging features of MIFS for its accurate diagnosis and appropriate management. However, histopathological diagnosis is ultimately required, and imaging-guided biopsy may be useful for target-enhancing solid areas of MIFS on magnetic resonance imaging (MRI).

Radiographs may be normal or show a non-specific soft tissue mass without calcification. Although osseous erosion or invasion has been reported in only a few cases [14,15,16,17], the underlying bone is usually normal. Ultrasonography reveals a hypoechoic mass with lobulated margins [17]. Color Doppler examination may display markedly increased vascularity. On MRI, MIFS usually shows a poorly circumscribed soft tissue mass with low to intermediate signal intensity in T1-weighted images and high signal intensity in T2-weighted images [15,16,17,18,19]. Surrounding edema reflecting the histological inflammatory component may also be seen. Contrast-enhanced MRI demonstrates homogeneous or heterogeneous enhancement [19]. The enhancement pattern in the majority of MIFSs is diffuse [17]. Tateishi et al. reported that extensive involvement adjacent to the tendon sheath was a common feature [18]. In our limited experience, the differentiation of MIFS from tenosynovitis can be challenging based on preoperative MRI features. Recently, Gaetke-Udager et al. reported one case of dedifferentiated MIFS and two cases with histologically high-grade areas [17]. The authors suggested that peripheral enhancement, a non-acral site, and a lack of association with the tendon might indicate high-grade foci or dedifferentiation. However, larger studies are required to verify these imaging features in MIFS.

## 4. Pathogenesis

MIFS is cytogenetically characterized by a balanced or unbalanced t(1;10)(p22;q24) translocation [20,21]. The same translocation has also been identified in hemosiderotic fibrolipomatous tumor (HFLT) [21,22,23], hybrid MIFS/HFLT [23,24], and pleomorphic hyalinizing angiectatic tumor (PHAT) [25], suggesting a pathogenetic link between these entities. In addition to the t(1;10) translocation, the presence of ring and/or marker chromosomes composed of material from chromosome 3 (3p11-12) has been shown in a small subset of MIFS cases [21,26]. Interestingly, ring and marker chromosomes have been associated with certain intermediate- or low-grade malignant mesenchymal tumors such as atypical lipomatous tumor/well-differentiated liposarcoma, dermatofibrosarcoma protuberans, and parosteal osteosarcoma [21]. Moreover, a balanced t(2;6)(q31;p21.3) translocation has been described as the sole anomaly in a single case [27].

Conventional comparative genomic hybridization (CGH), array CGH, and SNP array analyses have revealed the amplification of 3p11-12 [12,21,28,29,30]. This amplification is associated with the increased expression of the vestigial-like family member 3 (*VGLL3*) and charged multivesicular body protein 2B (*CHMP2B*) genes [21]. Notably, *VGLL3* amplification has been confirmed with fluorescence in situ hybridization (FISH) [23,31,32] and occurs in approximately half of all MIFS cases examined [12]. It is of interest that *VGLL3* amplification is also found in HFLT and hybrid MIFS/HFLT [23]. Recently, Klubíčková et al. detected a novel SEC23-interacting protein (*SEC23IP*)–*VGLL3* fusion in a single case lacking *VGLL3* amplification [32]. Gene fusions involving *VGLL3* have previously been reported in hybrid schwannoma/perineurioma [33] and spindle cell rhabdomyosarcoma [34]. The physiological role of VGLL3 is still poorly understood, but it is thought to function as a transcriptional cofactor by binding to TEA domain (TEAD)-containing transcription factors via the TONDU domain and has been shown to promote tumor cell proliferation through the activation of the Hippo pathway [35]. Moreover, in an undifferentiated sarcoma-derived cell line, VGLL3 has been shown to be required for proliferation [36]. In a recent study, it was uncovered that VGLL3 activates an inflammatory response by inducing interleukin-1α secretion [37]. These findings suggest that *VGLL3* is involved in the development and/or progression of MIFS.

In 2009, Hallor et al. investigated the breakpoint regions on chromosomes 1 and 10 in eight MIFS cases using FISH, array CGH, global gene expression profiling, and real-time quantitative polymerase chain reaction (PCR) [21]. The authors showed that the breakpoints in the t(1;10) translocation mapped to transforming growth factor beta receptor 3 (*TGFBR3*) in 1p22 and in or near O-GlcNAcase (*OGA*) in 10q24, resulting not in a functional fusion transcript but rather in an upregulation of nucleophosmin/nucleoplasmin 3 (*NPM3*) and fibroblast growth factor 8 (*FGF8*), two genes located close to *OGA*. Subsequent FISH studies by Antonescu et al. also demonstrated the presence of *TGFBR3* and *OGA* rearrangements in five (71%) of the seven pure MIFS cases, as well as in 12 (86%) of the 14 HFLT cases and all three hybrid MIFS/HFLT cases examined [23]. However, further FISH studies showed a very low percentage of *OGA* rearrangement in pure MIFS, present in 17% (1/6) or 6% (2/31) of cases [38,39]. In these two studies, *TGFBR3* rearrangement was not observed in any MIFS cases. Zreik et al. concluded that *TGFBR3* and/or *OGA* rearrangements are much more common in hybrid MIFS/HFLT than in classical MIFS [39]. Overall, t(1;10) *TGFBR3*/*OGA* rearrangement has been identified in 15 (13.4%) of the 102 MIFS cases examined [12].

A novel and recurrent genetic event—B-Raf proto-oncogene, serine/threonine kinase (*BRAF*) rearrangement/amplification—was recently detected in a subset of *TGFBR3*/*OGA*-negative MIFS cases but not in HFLT or hybrid MIFS/HFLT [31]. In that study, a target of myb1-like 2 membrane trafficking protein (*TOM1L2*)-*BRAF* fusion was identified in a single case. Since then, several *BRAF* fusion partners have been discovered in MIFS, including roundabout guidance receptor 1 (*ROBO1*) [30], zinc finger protein 335 (*ZNF335*) [12], staphylococcal nuclease and tudor domain-containing 1 (*SND1*) [32], and translocase of outer mitochondrial membrane 70 (*TOMM70*) [40]. It is of interest that *BRAF* abnormalities are mutually exclusive from *TGFBR3*/*OGA* rearrangements but can coexist with *VGLL3* amplification [31]. Overall, *BRAF* alterations have been identified in 11 (10.6%) of the 104 MIFS cases examined [12].

Most recently, Perret et al. showed the presence of a novel yes1-associated transcriptional regulator (*YAP1*)–mastermind-like transcriptional coactivator 2 (*MAML2*) fusion in three cases of nodular necrotizing MIFS [40]. This *YAP1*–*MAML2* fusion has previously been reported in a variety of tumor types, including poroma and porocarcinoma [41]; metaplastic thymoma [42]; retiform hemangioendothelioma and composite hemangioendothelioma [43]; atypical tenosynovial giant cell tumor [44]; spindle cell/sclerosing rhabdomyosarcoma [45]; and malignant undifferentiated epithelioid neoplasm [46]. However, its function is poorly understood. In addition, a novel TEA domain transcription factor 1 (*TEAD1*)–myocardin-related transcription factor B (*MRTFB*) fusion was detected in a single case lacking *VGLL3* amplification [32]. TEAD1 belongs to the family of TEAD proteins (from 1 to 4) that are key transcription factors in the Hippo pathway. Dysregulation of the Hippo pathway leads to aberrant cell growth and neoplasia [47]. Gene fusions involving *TEAD1* have previously been reported in congenital/infantile spindle cell rhabdomyosarcoma [48]. MRTFB is a transcription coactivator of the serum response factor. Recurrent gene fusions involving *MRTFB* have previously been demonstrated in chondroid lipoma [49] and ectomesenchymal chondromyxoid tumor [50].

The deletion of chromosome 13q is one of the most frequent genomic imbalances in MIFS [12,30]. Arbajian et al. reported that 14 out of the 256 genes in the commonly deleted region of 13q had a significantly lower expression, including abhydrolase domain-containing 13 (*ABHD13*), ALG11 alpha-1,2-mannosyltransferase (*ALG11*), arginine and glutamate rich 1 (*ARGLU1*), component of oligomeric golgi complex 3 (*COG3*), DnaJ heat shock protein family (Hsp40) member C3 (*DNAJC3*), G protein-coupled receptor 180 (*GPR180*), protein O-glucosyltransferase 2 (*POGLUT2*), muscleblind-like splicing regulator 2 (*MBNL2*), mediator complex subunit 4 (*MED4*), Nedd4 family-interacting protein 2 (*NDFIP2*), TDP-glucose 4,6-dehydratase (*TGDS*), transmembrane 9 superfamily member 2 (*TM9SF2*), transmembrane phosphoinositide 3-phosphatase and tensin homolog 2 pseudogene 5 (*TPTE2P5*), and UTP14C small subunit processome component (*UTP14C*) [30]. Moreover, it is of interest that RB transcriptional corepressor 1 (*RB1*), a well-known tumor-suppressor gene, is located on chromosome 13q14.2. The deletion of *RB1* has been described in a variety of soft tissue tumors [51,52]. Further studies are required to elucidate the biological consequences of these genomic alterations in MIFS.

## 5. Histopathology

Grossly, MIFS is lobulated and varies from gelatinous to firm or fleshy, often heterogeneous in color and texture. Hemorrhage and necrosis may be seen in high-grade MIFS.

Histologically, MIFS is typically multinodular and poorly circumscribed and shows alternating myxoid and fibrous/solid areas with a dense associated inflammatory infiltrate. The solid areas are mostly composed of sheets of round epithelioid cells or fascicles of spindle cells. One of the most distinctive histological features of MIFS is the presence of larger, atypical epithelioid cells that are often bi- or multinucleated and resemble Reed–Stenberg cells or virocytes. Pseudolipoblasts containing cytoplasmic mucin may be seen in the myxoid areas. The inflammatory infiltrate consists of lymphocytes, plasm cells, histiocytes, eosinophils, and neutrophils. Mitotic activity is minimal, and necrosis is uncommon [4]. Given its heterogeneous histological features, MIFS may be mistaken for other soft tissue tumors with a myxoid or inflammatory background, including superficial acral fibromyxoma, low-grade fibromyxoid sarcoma, myxofibrosarcoma, and inflammatory myofibroblastic tumor.

In 2015, Michal et al. reported 23 cases of high-grade MIFS [7]. In contrast to conventional MIFS, high-grade MIFS exhibits increased cellularity and mitotic activity. Additionally, atypical mitoses are common, and tumor necrosis is variably present. One of the most characteristic hallmarks of high-grade MIFS is the presence of emperipolesis [7]. Emperipolesis is much easier to find with the help of immunohistochemistry [7]. At least focally, however, areas of conventional MIFS with a myxoid and inflammatory background are present.

Most recently, Perret et al. described seven cases of nodular necrotizing MIFS, with a good prognosis compared with conventional MIFS [40]. This distinctive variant of MIFS commonly occurs in the extremities and is characterized by frequent nodular configuration, a marked predominance of Reed–Stenberg cells/virocytes, a lack or very focal presence of myxoid stromal changes, and the presence of central necrosis. A moderate to dense inflammatory infiltrate can be present. Mitotic activity is generally low, ranging from 1 to 9 mitoses per 10 high-power fields, and atypical mitoses are rarely seen. As mentioned above, a *YAP1–MAML2* fusion has been identified in nearly half of cases. The histological variants are summarized in Table 1.

Immunohistochemically, the tumor cells are diffusely positive for vimentin and focally positive for CD68 and CD34. Recent immunohistochemical studies demonstrate strong expressions of bcl-1 (94.5%), factor XIIIa (89%), CD10 (80%), and D2-40 (56%) [12]. Immunostainings for CD15, CD30, CD45, CD99, CD117, S-100 protein, HMB-45, desmin, calponin, and CAM5.2 are generally negative. The nodular necrotizing variant of MIFS may show the focal expression of cytokeratin AE1/AE3 [40]. Immunostaining for cyclin D1 is recommended to identify emperipolesis for conventional MIFS [7]. Moreover, high-grade MIFS can show preferentially expressed antigen in melanoma (PRAME) immunoexpression [7,8]. The MIB1 labeling index is typically low (less than 1–2%).

Two other soft tissue tumors, HFLT and PHAT, share some histological features with MIFS, and all three have been found to possess a t(1;10) translocation. Moreover, the neoplastic cells in these tumors have a similar immunophenotype. HFLT is a locally aggressive neoplasm of intermediate malignancy that typically arises in the subcutaneous tissue of the foot or ankle in middle-aged women [53]. Histologically, HFLT consists of variable distributions of mature adipocytes, spindle cells with intracytoplasmic hemosiderin, hemosiderin-laden macrophages, and osteoclast-like giant cells. HFLT may show stromal myxoid changes and a mixed chronic inflammatory infiltrate. Mitotic activity is low, and necrosis is absent. Several cases showing mixed features of HFLT and conventional MIFS have been reported [23,24,38,39]. Immunohistochemically, the spindle neoplastic cells are positive for CD34 and calponin [53]. PHAT is a locally aggressive but non-metastasizing neoplasm that usually occurs in the subcutaneous tissue of the lower extremities in middle-aged to older adults [54]. Histologically, PHAT is characterized by clusters of variably sized, thin-walled, ectatic blood vessels, often containing organizing thrombi and surrounded by a thick rim of amorphous eosinophilic material. The neoplastic cells include spindled to oval fibroblastic cells and pleomorphic cells with frequent nuclear pseudoinclusions. A mixed chronic inflammatory infiltrate is often present. Mitotic activity is very low, and necrosis is absent. PHAT may show peripheral areas with features identical to those of HFLT [55]. Immunohistochemically, the neoplastic cells are typically positive for CD34 [54]. Recently, Michal et al. suggested that most if not all tumors diagnosed as a PHAT may represent examples of MIFS that, in addition to a conventional MIFS histology, manifest aberrant ectatic hyalinizing blood vessels [56]. On the other hand, Boland and Folpe have proposed that HFLT is the early stage of PHAT, whereas MIFS is not related to either HFLT or PHAT [57].

In our opinion and experience, the most significant differential diagnosis is myxofibrosarcoma. Myxofibrosarcoma is one of the most common soft tissue sarcomas (STSs) that typically arise in the subcutaneous tissue of the extremities in older adults. In general, myxofibrosarcoma is cytogenetically associated with highly complex karyotypes lacking specific chromosomal abnormalities [58]. Histologically, myxofibrosarcoma can be subdivided into three grades on the basis of the degree of cellularity, nuclear pleomorphism, and proliferative activity [59]. Low-grade myxofibrosarcoma is composed of spindle cells with mildly atypical, hyperchromatic nuclei in a variably myxoid stroma. Mitotic activity is low, and necrosis is absent. Intermediate-grade myxofibrosarcoma is more cellular and pleomorphic than purely low-grade myxofibrosarcoma and often contains minute solid areas showing flank pleomorphism. High-grade myxofibrosarcoma consists of severe atypical spindle cells and bizarre, pleomorphic giant cells. Atypical mitoses are common, and necrosis is variably present. In contrast to MIFS, elongated, curvilinear, thin-walled blood vessels are present in myxofibrosarcoma. The Reed–Stenberg-like cells or virocytes seen in MIFS are absent, although pseudolipoblasts can be found. Immunohistochemically, the tumor cells are occasionally positive for smooth muscle actin and CD34 [59]. Immunostainings for S-100 protein and desmin are typically negative. The detection of *TGFBR3* and/or *OGA* rearrangements caused by FISH can serve to distinguish MIFS from myxofibrosarcoma.

## 6. Management

### 6.1. Localized Disease

Surgical resection is the standard treatment for local disease. The standard surgical procedure is wide resection with negative margins (R0, no residual microscopic tumor). However, resection with R0 margins is more challenging for MIFS given its infiltrative growth. In selected cases, amputation may be an option when wide resection fails to preserve limb function [11]. Recently, Fujiwara et al. reported that excellent local control in low-grade STS was achieved with microscopic margins greater than 2 mm [60]. We recommend a minimum 2 cm margin width for the resection of infiltrative STSs, including MIFS. It should be kept in mind that the rate of local recurrence for MIFS in R0 resection is relatively high compared with other low-grade STS subtypes.

Radiotherapy (RT) can be used as a neoadjuvant/adjuvant treatment strategy to improve local disease control. Although the role of RT in the management of MIFS remains debatable, Tejwani et al. reported that RT in combination with surgery is associated with a lower risk of local recurrence [13]. In this study, despite 5 of the 14 patients treated with preoperative RT having positive surgical margins after initial resection, all of these patients were without evidence of local recurrence at the final follow-up. No late RT toxicity higher than grade 2 was identified in the radiated patients. Laskin et al. also reported that four of the six patients receiving RT after re-resection of recurrent disease were disease-free for over 5 years, and one was free of additional disease for 12 months [9]. Further prospective randomized trials are needed to better define optimal treatment approaches for localized MIFS.

### 6.2. Advanced/Metastatic Disease

The development of unresectable locally advanced or metastatic MIFS is associated with a poor prognosis [7,29,61,62]. Currently, there is no regulatory-approved treatment for advanced/metastatic MIFS.

There are several case reports concerning the systemic treatment of patients with recurrent/metastatic MIFS [7,9,29,61,63]. Laskin et al. reported an atypical MIFS of the ankle that developed multiple recurrences and metastases [9]. The patient was placed on imatinib mesylate after the last thigh recurrence and was disease-free for 59 months. Fagerstedt et al. presented an aggressive MIFS of the foot with metastatic spread and a fatal outcome within 16 months [29]. Combination chemotherapy with an IVADIC (ifosfamide, vincristine, doxorubicin, and dacarbazine) regimen was started after recurrence, but the response was poor. After that, the patient had a widespread disease with metastases in the lung and retroperitoneum, and no response to treatment with etoposide was obtained. Sparkman et al. described a high-grade, aggressive MIFS of the calf that progressed to the patient’s death in less than 2 years despite multiple therapies [61]. The patient received adjuvant therapy with gemcitabine and docetaxel after the re-resection of the recurrent disease and RT, but the response was poor. These results should be interpreted with caution because the number of cases is too small to make any definitive conclusion.

Several solid tumors with BRAF fusions have shown evidence of clinical sensitivity to RAF or MEK inhibitors on the basis of a few in vitro and clinical studies [64,65,66,67]. Interestingly, Ross et al. reported a case of malignant spindle cell tumor of the chest wall with a *BRAF* fusion that responded to treatment with an oral multikinase inhibitor sorafenib in combination with bevacizumab and temsirolimus [67]. As noted above, BRAF fusions have been identified in a subset of MIFSs. Based on these findings, we speculate that RAF or MEK inhibitors may be an effective therapeutic option for advanced/metastatic MIFS featuring BRAF fusions.

Immunotherapy is an emerging treatment for several cancer types with promising outcomes, including cancer vaccines, adoptive cellular therapies, and immune checkpoint inhibitors [68]. The major targets of FDA-approved immunotherapeutic antibodies are programmed cell death protein-1 (PD-1) and its ligand, programmed cell death ligand-1 (PD-L1). The PD-1/PD-L1 interaction is a major pathway hijacked by tumors to suppress immune control. The expression of PD-L1 has recently been reported in intermediate (locally aggressive) soft tissue tumors such as desmoid-type fibromatosis [69]. Unlike chemotherapy, immunotherapy relies on stimulating the natural defenses of the host immune system to attack malignant cells. Most recently, Pulvers et al. reported a PRAME-positive high-grade MIFS of the neck that progressed to multi-side metastatic disease despite adjuvant therapy [8]. It is of interest that PRAME expression was seen only in the high-grade areas. Michal et al. also reported that immunopositivity for PRAME was found in most high-grade MIFS cases [7]. PRAME is a cancer–testis antigen that is expressed in the normal testis and several sarcoma subtypes, such as synovial sarcoma, multifocal leiomyosarcoma, myxoid/round cell liposarcoma, and osteosarcoma [70,71,72]. Moreover, PRAME expression is associated with poor prognosis in STS [72]. It is important to note that PRAME has emerged as a potential candidate target for immunotherapy. Clinical trials of cellular immunotherapy-targeting PRAME are currently ongoing in rhabdomyosarcoma (NCT02239861) and solid tumors (NCT02789228) [73]. Results from these trials are eagerly anticipated.

## 7. Conclusions and Future Directions

MIFS is a unique form of ultra-rare sarcoma that typically arises in the subcutaneous tissue of the distal extremities in middle-aged adults and has a high propensity for local recurrence. Histologically, MIFS is characterized by distinctive virocytes or Reed–Stenberg-like cells and is associated with a prominent mixed inflammatory infiltrate. Notably, it should be kept in mind that high-grade MIFS behaves more aggressively. MIFS displays a recurrent t(1;10)(p22;q24) translocation, with rearrangements of *TGFBR3* and/or *OGA*. *VGLL3* amplification is the most consistent alteration, seen in roughly 50% of cases. Surgical resection is the mainstay of treatment for localized MIFS, although the use of RT in combination with surgery may be considered in appropriately selected patients. The management of advanced/metastatic MIFS remains challenging. It is often difficult to carry out robust research/clinical trials in ultra-rare sarcomas like MIFS; therefore, a sustainable global collaborative effort is required for improvement in the clinical outcomes of advanced/metastatic MIFS in the future.

## Figures and Tables

**Table 1:** Histological variants of MIFS.

Histology	Cellularity	Nuclear Pleomorphism	Mitotic Activity	Necrosis
Nodular necrotizing	Low to moderate	Rare	Low	Central
Conventional	Low to moderate	Rare	Low	Absent
High-grade	High	Pronounced	High	Frequent

## Data Availability

Not applicable.
